# COVID-19 Impact on Teachers’ Organizational Commitment in Schools

**DOI:** 10.3389/fpsyg.2022.810015

**Published:** 2022-05-31

**Authors:** Izlem Şerife Safkan Akartuna, Oğuz Serin

**Affiliations:** Faculty of Education, European University of Lefke, Lefke, Turkey

**Keywords:** teachers’ commitment, COVID-19 pandemic, online education, school lockdown, digitalisation

## Abstract

Highly committed teachers spend more effort helping their schools achieve their academic goals. The COVID-19 pandemic had a dire effect on education worldwide. However, just after a few semesters, teachers were asked to return back to schools to teach in person. This study aims to analyze the organizational commitment levels of school teachers before and after the implementation of the COVID-19 pandemic measures that resulted in a two-semester break in face-to-face teaching. In this study, a quantitative research method was utilized. Commitment levels of a total of 300 teachers were assessed in a longitudinal test by using a popular tool in order to see the relative change as a result of the COVID-19 pandemic in Cyprus. The study mainly focuses on gender, marital status, education levels, job experience, and duration of work in the organizations, and the information obtained in the form of responses was statistically analyzed. According to the results of the quantitative analysis, the sampling average for the organizational commitment was determined to be at a medium-to-high level. The commitment levels of teachers were observed to be decreased after the COVID-19 pandemic measures came to be implemented in schools. A detailed investigation of the school teachers’ commitment levels corresponding to the different demographic characteristics before COVID-19 and after the implementation of normalization measures is presented in this study.

## Introduction

The dynamic changes that influence societies can alter the social characteristics of individuals and disciplines. The COVID-19 pandemic resulted in the abrupt transition from school education to online distance education, with teachers being left with no time to make any preparation. Several studies were conducted on the difficulties the school teachers faced during the online education period. According to the UNESCO report (UNESCO, 2021), despite the efforts made for online education, more than 500 million people were excluded from access to education. For example, the difficulty in accessing the internet is considered as the primary concern pertaining to access to online education (Giannini, 2020). These difficulties brought new responsibilities to teachers, which came to be added to their current workload (Correia, 2020). As a result of the pandemic measures taken at schools, the social characteristics of teachers are expected to be influenced by the additional load. According to a study by Kieschke and Schaarschmidt (2008), the commitment levels of teachers were monitored to be affected by anxiety about health conditions. Also, school teachers were observed to show reduced commitment to their organizations during the online teaching period, and this was associated with increased personal stress (Malik, 2020).

An organization’s social structure has a sensitive balance, and there is more than one component affecting this structure. Barnard (1938) defines an organization as “a system of purposefully coordinated actions or powers of two or more individuals.” For organizations to achieve their quality goals, they should create structures and relationships to ensure that the individuals constituting the organization unite around a goal and concentrate on that goal (İra and Bulut, 2018).

While there have been several studies that have assessed online education and the problems faced by teachers during the online education period caused by the pandemic, there are only limited studies focusing on face-to-face education as the pandemic continues. This study focuses on the organizational commitment levels of school teachers before the COVID-19 pandemic lockdown and during the first face-to-face education period, which was initiated as a part of the normalization measures implemented in Cyprus.

## Literature Review

### Organizational Commitment

There is a consensus that organizational commitment involves a direct relationship between the employees and their organizations. However, there has been a divergence in the structure and formation of this relationship. This divergence is reflected in the existence of various definitions of organizational commitment, which has further resulted in the emergence of different definitions (Gül, 2002). Organizational commitment has been the subject of discussion in various studies for over 50 years (Allen and Meyer, 1990; Karrasch, 2013).

Organizational commitment is addressed as a three-component concept by Meyer et al. (1993). According to this model, organizational commitment is made up of three different elements: (1) emotional (affective) commitment, (2) continuity commitment, and (3) normative commitment. These components have been confirmed by different studies (Allen and Meyer, 1996). The most adopted commitment classification in literature is the one created by Allen and Meyer (1996). As per their approaches, including the three-component organizational commitment models, commitment is a psychological state (Meyer et al., 1993). This model expresses the characteristics of an employee’s relationship with the organization and the signs determining the continuation of the employee’s association with the organization (Güçlü, 2003). Organizational commitment can be defined as the level of an employee’s embracement of the goals and norms of the organization, emotional commitment felt toward the organization, and the willingness to continue to work in that organization (Allen and Meyer, 1996).

Affective commitment occurs as a result of an individual’s positive attitude toward an organization; it is the combination of the individual’s work experiences, perceptions, and personal characteristics (Mowday et al., 1982). Cengiz (2002) suggests the affective commitment to be a type of commitment that includes not only complying with the general functioning of the organization within the framework of the rules determined by the organization, but also participating in all the activities of the organization sincerely to develop it, improve its existence in the best way, and make it superior against its competitors.

Continuance commitment is related to an employee’s desire to stay in the organization because of the rewards gained by staying in the organization or the loss of leaving the organization (Balay, 2000; Kalay, 2015). If the person has a high continuance commitment level, it may not be necessarily related to the joy he/she draws from the job. If the continuance commitment level dominates the general commitment level, the reason can be associated with the individual benefits the individual gets due to the job (Özkalp and Kirel, 2013).

An employee feels morally obliged to stay in the organization when the normative commitment levels are high. It is suggested by Meyer and Allen (1991) that an employee feels obliged to stay in the organization due to the feeling of responsibility (Wasti, 2002).

Teachers’ perceptions of the quality of the school environment affect their organizational commitment and in turn affect their job performance and the education they provide in school (Hoy and Miskel, 1987). The study by Hoy et al. (1990) suggests that affective schools are possible with teachers having high commitment levels. In addition, demographic characteristics can play a huge role in commitment levels, as suggested by Özkaya et al. (2006).

Schools globally were forced to provide online education due to the COVID-19 pandemic. During the COVID-19 online education period, teachers faced increased stress levels due to the increased scope of responsibilities (e.g., housework and unprepared online education), as suggested by Cigerci (2020) and MacIntyre et al. (2020). As per the study of Hoang (2020), teachers faced social and financial problems during the online education period.

Although several studies point out the difficulties that occurred during the online teaching period in the COVID-19 pandemic, there is no comparative study that focuses on teachers’ commitment levels before and after the COVID-19 pandemic. This study aims to investigate the organizational commitment levels of teachers, in accordance with the answers provided by the teachers to quantitative questions and bring out the problems faced by them.

In particular, this study will first investigate the teachers’ perception of organizational commitment and will ask if the teachers’ levels of organizational commitment levels change with the gender variable, marital status, education level, self-development, job experience, and the total time spent in the same organization. The second research question will ask if the teachers’ commitment levels changed as a result of COVID-19 precautions that were implemented at schools in 2020.

## Method

### Research Model and Sampling

This study aims to research the organizational commitment of teachers working in secondary schools under the North Cyprus Ministry of Education. It was carried out by using the relational screening model. A research study investigating relationships and bonds is mainly called relational screening (Büyüköztürk et al., 2008). Determining the demographic characteristics of teachers and the relationship between the changes in the commitment levels before and after a pandemic forms the main focus.

Data were collected from the survey forms shared with 300 teachers working in different educational institutions, chosen by quota sampling. The data collection took place on the Teachers Union online communication portal by communicating with a group of teachers selected on a non-proportional quota sampling basis. The method was implemented in such a way that all groups have minimum participants contributing to the survey. Online forms were delivered through the Teachers Union mail list where the data collection was carried out twice on the same group of teachers, first during the 2018–2019 education year and the other was during the post-pandemic 2021–2022 face-to-face education year. A total of 2,055 teachers working in 32 schools, consisting of 14 elementary and 18 high schools, under the Education Department of Northern Cyprus constitute the sample population of this study. The participants are about 15% of the total teacher population, which corresponds to approximately a 5% confidence interval at a 95% confidence level. In the study, 58% of the participants were women and 42% were men. About 40% of the participants had more than 16 years of experience, and 82% of the sample population was married. About 66% of the participant teachers had bachelor’s degree, and the rest of the participants had master’s degree as the final education level.

### Study Design

Organizational commitment levels were investigated under three main components: affective, continuance, and normative. The commitment levels specific to demographic characteristics of teachers were assessed before and after the COVID-19 measures were implemented at schools. The main idea of this longitudinal study is to find the most influenced group of teachers as a result of the COVID-19 pandemic and the relative reduction in their commitment levels.

### Instruments

The organizational commitment scale developed by Meyer et al. (1993), translated into Turkish by Dağli et al. (2018), was used in this study to evaluate the commitment levels of teachers. With the help of the translated version, the tool was applied to Turkish-speaking teachers for several studies. Reply options are graded using the 5-point Likert scale with the following options: “Completely agree,” “Agree,” “Indecisive,” “Disagree,” and “Completely disagree.” In positive items, “Completely agree” is given 5 points, and “Completely disagree” is given 1 point. The tool uses 18 questions, where the overall minimum grading is 18, showing the lowest commitment level, and the overall maximum grade is 90, indicating the highest commitment level (considering some questions are graded in reverse format). The quantitative tool to be used in the research were applied using the online form for the teachers working in the schools chosen for the sample collection, and details are given in data collection procedures. In the first phase of the statistical study that was to be carried out in two phases, an organizational commitment was applied. Microsoft Excel Data Analysis ToolPak (Microsoft Corporation, 2018) was used to carry out the analysis. ANOVA and *t*-tests were performed to identify any relationship that is available in each demographic class. In total, two surveys were completed to collect certain demographic information. The statistical distribution of the investigated demographic information is provided in Table 1.

**TABLE 1:** Distribution of demographic variables in the sample.

Demographic variable	Category	Participant, %
Gender	Female	58
	Male	42
Marital status	Married	82
	Single	18
Work experience	1–5 Years	15
	6–10 Years	16
	11–15 Years	29
	16+ Years	40
Time spent in same school	1–5 Years	31
	6–10 Years	15
	11–15 Years	24
	16+ Years	29
Education level	Bachelor degree	66
	Master’s degree	34
Type of school	Secondary school	53
	High school	26
	College	21
Professional development	Active	47
	Passive	53

The reliability test was conducted for the applied organizational commitment survey, and Cronbach’s alpha values for the post-tests were determined to be 0.802 (0.781 for affective commitment, 0.832 for continuance commitment, and 0.821 for normative commitment) for the pre-COVID period. On the other hand, post-COVID period, Cronbach’s alpha values for the post-tests were found to be 0.852 (0.841 for affective commitment, 0.839 for continuance commitment, and 0.821 for normative commitment). The probability significance value (Bartlett’s test) for both applications was less than 0.05, which allowed the sample to be considered in the target population. In this regard, the male-to-female ratio of gender distribution within the sample was seen to be close.

### Data Collection and Analysis

Data were collected from the survey forms shared with the 300 teachers working in different educational institutions, which were chosen *via* minimum participant condition non-proportional quota sampling method. The quota sampling method is one of the non-probability sampling methods that allow the researcher to limit the target audience in terms of certain characteristics (Cohen et al., 2008). Two simultaneous conditions were used in recruiting participants. First, the whole number of participants should constitute a minimum of 15% of all teachers currently working in the Education Department of Northern Cyprus. The participants are about 15% of the total teacher population, which corresponds to approximately a 5% confidence interval at a 95% confidence level. However, as the general sample size is small and several demographic variables exist in parallel, instead of using a random method and risking the involvement of small groups, an additional condition was utilized in the sample selection for securing the contribution of each demographic sub-group. Based on the approach, each sub-group was required to form a minimum of 15% of the sample. The data collection was carried out twice, first during the 2018–2019 education period, and the other during the post-pandemic 2021–2022 face-to-face education period. In the first section of the survey, teachers’ personal information was collected. Then in the second section, the organizational commitment scale developed by Meyer et al. (1993), translated into Turkish by Dağli et al. (2018), was used. This tool is considered to be a strong tool, as it is well-suited for teachers working at schools in Turkey. Once the data collection is complete, the survey results were evaluated to check if the data are suitable for normal distribution. Skewness was found to be −0.22 before the COVID-19 period and −0.27 after the COVID-19 period. Similarly, the Kurtosis coefficient was also studied, where the coefficient was found to be 1.05 before the COVID-19 period and 0.61 after the COVID-19 period. Additionally, Kolmogorov–Smirnov test was conducted on independent test groups by using MATLAB Mathworks (2015). The significance level was considered 0.05 in the study based on the test results, and normal distribution was verified for the independent group tests. Once the data were verified, the overall average test results of demographic variables were first compared. Then *t*-tests and ANOVA tests were conducted on data where applicable to determine the significant differences between the commitment levels of groups.

## Findings

The results of the organizational commitment survey conducted before the COVID-19 pandemic and after the lockdown measures ended are summarized in Table 2. Before the COVID-19 pandemic, the mean (X) result for organizational commitment was 3.57, and after the lockdown measures ended, the mean result (X) for organizational commitment was 3.24. When the subcomponents were investigated for the pre-pandemic condition, it was seen that the highest level subcomponent was affective commitment (X: 4.18, S: 0.90), followed by continuity commitment (X: 3.47, S: 1.24) and normative commitment (X: 3.04, S: 1.42). On the other hand, a significant reduction was observed when the commitment levels were monitored following the end of the school lockdown. When compared to before the COVID-19 school lockdown period, the affective and continuance commitment subcomponents showed a reduction in mean properties. The affective subcomponent reduced by 10%, and the continuance commitment levels dropped by 18%. The change in normative commitment levels was negligible. The variance between the participant answers was higher in the post-pandemic period. This indicates a non-homogeneous influence over the demographic groups. Also, the significant difference between each subcomponent before and after pandemic conditions was investigated through *t*-tests. A significant difference at a 0.05 level was observed for the affective and continuance commitment subcomponents with change in time. When effect size was studied for both affective and continuance commitment levels, Cohen’s *d*-value of 0.34 was observed for affective commitment change and 0.46 was observed for the continuance commitment change. Both results are in the range of small-to-medium effect size of practical significance.

**TABLE 2:** Subcomponent commitment levels of teachers (after Meyer et al., 1993).

	Before/after COVID-19 lockdown	Mean, X	Standard deviation, S	*t*	*P* *
Affective commitment	Before	4.18	0.90	1.85	0.01*
	After	3.78	1.44		
Continuance commitment	Before	3.47	1.24	2.56	0.00**
	After	2.85	1.48		
Normative commitment	Before	3.04	1.42	1.16	0.92
	After	3.08	1.28		

*P < 0.05, **P < 0.01.

In addition to the global comparison, demographic characteristics were also considered to carry out the comparison. A *t*-test was conducted for the classification with respect to gender, marital status, education level, and self-development. The results are presented in Table 3. In situations where the female participants’ commitment levels were observed to reduce by 12%, the male participants’ commitment levels were observed to only drop by 2%. When the marital status of the participants and the corresponding change in their commitment levels were studied, a slight decline was observed in the commitment levels of married teachers. However, the commitment levels of single teachers slightly increased as a result of the investigation which took place before and after the COVID-19 school lockdowns. The education level of the teachers was also monitored to influence the change in commitment levels. Although the change in commitment level was insignificant in the case of teachers with a master’s degree, the teachers with a bachelor’s degree showed an 18% reduction in their commitment levels.

**TABLE 3:** *T*-test results of organizational commitment levels of teachers before and after COVID-19 lockdown.

		Mean, X	Std. Dev., S	*t*	*P* *
Before	Female	3.58	0.57	1.65	0.38
	Male	3.56	0.55		
After	Female	3.16	1.23	1.58	0.07
	Male	3.48	1.12		
Before	Married	3.59	0.57	1.68	0.01*
	Single	3.40	0.44		
After	Married	3.43	0.27	0.95	0.18
	Single	3.68	0.52		
Before	Bachelor degree	3.66	0.58	1.65	0.000**
	Master‘s degree	3.40	0.48		
After	Bachelor degree	2.99	0.37	1.72	0.04*
	Master‘s degree	3.32	0.31		
Before	Active at Continuing Professional Dev.	3.60	0.50	1.97	0.45
	Passive at Continuing Professional Dev.	3.55	0.61		
After	Active at Continuing Professional Dev.	3.33	0.30	0.54	0.29
	Passive at Continuing Professional Dev.	3.21	0.57		

*P < 0.05, **P < 0.01.

Individual *t*-tests were applied to each demographic variable before and after pandemic conditions (Table 3). A significant reduction in the specific commitment levels was recorded in women due to the COVID-19 pandemic. Moreover, a significant relationship was observed in the group including the married teachers and the group consisting of teachers with bachelor’s degrees before and after COVID-19 conditions. On the other hand, teachers who are male or single or hold master’s degrees individually showed no significant relationship before and after COVID-19 periods.

A significant relationship was monitored for the variables, such as education level, work experience in general/experience in the same school, and between the school types. The results with regard to the effect of job experience and its components are summarized in Table 4. The results showed that the highest level of commitment (X: 3.86) was found in the groups which included teachers with 1–5 years of job experience. These values decrease as the job experience increases. However, the commitment levels of teachers with 16+ years of job experience occupied second place before the COVID-19 school lockdown period. This group showed a 15% reduction in the commitment level when monitored after the COVID-19 school lockdown period.

**TABLE 4:** ANOVA test results.

			Mean, X	Std. Dev., S	*F*	*P* *
Work experience	Before	1–5 Years	3.86	0.40	2.92	0.03*
		6–10 Years	3.58	0.31		
		11–15 Years	3.48	0.49		
		16+ Years	3.58	0.62		
	After	1–5 Years	3.81	0.49	8.56	0.005**
		6–10 Years	3.62	0.35		
		11–15 Years	3.36	0.42		
		16+ Years	3.06	0.72		
Time spent in same school	Before	1–5 Years	3.46	0.46	6.05	0.000**
		6–10 Years	3.70	0.45		
		11–15 Years	3.44	0.62		
		16+ Years	3.74	0.59		
	After	1–5 Years	3.42	0.50	9.66	0.000**
		6–10 Years	3.54	0.33		
		11–15 Years	2.98	0.37		
		16+ Years	2.89	0.68		
School type	Before	Secondary school	3.50	0.57	11.05	0.000**
		High school	3.54	0.47		
		College	3.92	0.53		
	After	Secondary school	3.01	0.41	3.45	0.04*
		High school	3.43	0.38		
		College	3.62	0.37		

*P < 0.05, **P < 0.01.

The commitment levels of teachers were classified in accordance to the school type as the secondary school (X: 3.50) and high school (X: 3.54) teachers, and the results were found to be similar between the groups. However, the commitment levels observed for the college teachers’ group were 12% higher in the pre-pandemic conditions. When the commitment levels were rechecked after the end of the COVID-19 school lockdown, a reduction of 3, 8, and 14% was observed for the high school, college, and secondary school teachers, respectively.

Individual *t*-tests were applied on before and after conditions of each demographic variable (Table 5). Significant relationship was recorded on female specific commitment levels reduction due to the Covid-19. Moreover, the married teachers group and teachers who hold Bachelor degree group showed significant relationship when before and after Covid-19 conditions were examined. On the other hand, teachers that is Male or Single or holds Master’s Degree individually showed no significant relationship with the before and after Covid-19 periods.

**TABLE 5:** Individual *t*-tests on demographic characteristics before and after COVID-19 pandemic conditions.

	Male	Female	Single	Married	Bachelor degree level	Masters degree level	Active at Continuing Professional Dev.	Passive at Continuing Professional Dev.
*t*	1.55	1.43	1.56	1.65	1.78	1.98	1.58	1.52
*P*	0.25	0.000**	0.15	0.015*	0.000**	0.09	0.10	0.06

*P < 0.05, **P < 0.01.

A *post-hoc* test was conducted for the variables to identify which category showed significant differences among groups of teachers with experience, being in the same school, and school type. When the test was conducted both before and after the COVID-19 periods, the trends of significant difference mostly remained the same among the groups. Table 6 summarizes the results of the *post-hoc* study where specific analysis was conducted on individual groups. Based on the *post-hoc* analysis, the significant difference was noticed only in those groups where the difference in experience years was more than 5 years. Furthermore, when the time spent in the same institution was investigated, a significant difference was observed between the groups having an experience of less and greater than 10 years. Finally, when school types were investigated, a significant difference was only observed between the college teachers and secondary school teachers during the post-COVID-19 period. On the other hand, a significant difference exists for any combination with secondary school teachers before the COVID-19 period.

**TABLE 6:** *Post-hoc t*-test results.

Work experience (years)							

		1–5 vs. 6–10	1–5 vs. 11–15	1–5 vs. 16 +	6–10 vs. 11–15	6–10 vs. 16+	11–15 vs. 16+
Before	*t*	3.21	3.12	3.52	3.55	3.62	3.75
	*P*	0.09	0.01*	0.00*	0.06	0.04*	0.05*
After	*t*	2.02	2.02	2.02	2.02	2.02	2.02
	*P*	0.08	0.00*	0.00*	0.08	0.01*	0.22

**Time spent in the same school (years)**							

		**1–5 vs. 6–10**	**1–5 vs. 11–15**	**1–5 vs. 16+**	**6–10 vs. 11–15**	**6–10 vs. 16+**	**11–15 vs. 16+**

Before	*t*	2.05	2.13	2.15	2.35	2.85	2.32
	*P*	0.18	0.01*	0.00*	0.00*	0.00*	0.06
After	*t*	2.02	2.02	2.02	2.01	2.05	2.04
	*P*	0.20	0.03*	0.01*	0.00*	0.00*	0.23

**School type**							

		**Secondary vs. High school**	**Secondary vs. College**	**High school vs. College**			

Before	*t*	2.12	2.21	2.22			
	*P*	0.02*	0.01*	0.82			
After	*t*	2.09	2.09	2.14			
	*P*	0.10	0.04*	0.67			

*P < 0.05, **P < 0.01.

The Pearson’s R correlation study was also carried out on the subcomponents of commitment, and the results are presented in Table 7. Results show no strong level of relationship between the subcomponents during the pre- and post-COVID periods. On the other hand, while obtaining no significant relationship when repeating the analysis between commitment subcomponents for the pre-COVID period, a medium-level relationship between affective and continuance subcomponents was observed for the post-COVID period. Although no positive strong relationship was found between the affective and normative subcomponents in the pre-COVID period, the negative correlation properties were replaced with positive values in the post-COVID period.

**TABLE 7:** Pearson’s R correlation test results.

		Affective	Continuance	Normative
Before	Affective	1.00		
	Continuance	0.34	1.00	
	Normative	−0.20	0.26	1.00
After	Affective	1.00		
	Continuance	0.45	1.00	
	Normative	0.31	0.11	1.00

## Discussion

COVID-19 influenced the field of education in various aspects. Recently, several studies were conducted to highlight the difficulties that the teachers faced during the COVID-19 school lockdown. This study assessed the relative changes in the organizational commitment levels of teachers before and after the COVID-19 school lockdown. Based on the studied parameters, there is an overall decrease in the commitment levels of teachers after the COVID-19 school lockdown. As suggested by Choi and Tang (2009), the commitment levels of teachers may change with a change in time. In this study, the relative decrease was observed in several parameters, such as demographics, education, and experience levels. Based on the answers of the teachers classified in accordance to the “time worked at the same school,” it was observed that the highest commitment was noticed in the 16+ years of experience group before the advent of pandemic conditions. These results are in line with the study of Collie et al. (2011). As per the respective study, a gradual increase in the commitment levels of teachers is expected with an increase in their experience. However, when the post-lockdown conditions were studied, the commitment levels dropped significantly in the group of teachers who had spent more than 10 years at the same school. In other words, the trend is now in the reverse direction. The negative impact of this trend can be associated with the feeling of discomfort among the experienced teachers (belonging to the higher risk group by age), as they are under the perception that the COVID-19 pandemic is still pernicious. Unlike the studies conducted in Turkey (Selvitopu and Şahin, 2013), this study on Turkish Cypriot teachers showed that the teachers with 16+ years of job experience occupied second place, showing that there is a decrease in the commitment levels of the teacher group with 5–16 years of job experience in the pre-COVID period. Brimeyer et al. (2010) suggest the more experience the worker has, the greater the organizational commitment levels. The idea comes from the high levels of autonomy of the experienced teachers and the greater control at the point of production. However, this was not true in the post-COVID period, where old and experienced teachers had only limited experience in online teaching portals.

The groups were evaluated in terms of the subcomponents of organizational commitment based on marital status, education level, job experience, and service time in the same school and school type. The COVID-19 pandemic has caused changes in several known facts and social characteristics of people. This study attempted to investigate the impact of COVID-19 school lockdown on the commitment levels of teachers. In general, the findings show that the commitment levels of teachers dropped in the range of 5–25%. The drop in commitment levels was more prominent in the female teachers when compared to the male teachers. Interestingly, the commitment levels of single teachers slightly increased. As suggested by MacIntyre et al. (2020), married teachers saw an increase in their responsibilities during the pandemic (e.g., simultaneous education with kids at home), which resulted in them having increased stress levels.

In general, the normative commitment levels of teachers were not significantly affected. However, their affective and continuance commitment levels dropped. Some teachers were reported to have retired early in order to avoid face-to-face teaching during the pandemic. Wei et al. (2021) suggest a reduction in the commitment levels of teachers due to the measures taken on teachers, such as salary reduction and changes in the work environment. Each demographic group showed a reduction in the commitment levels of teachers with a different pattern. Groups that showed significant differences were highlighted as a part of this study. However, due to the limitations of this study, no further reasoning could be carried out.

Furthermore, the effects of the pandemic on the commitment levels of teachers were different for different groups. COVID-19 pandemic resulted in the utilization of technology for education. Globally, teachers were forced to use digital tools to teach without any initial preparation. While some groups of teachers easily adapted to online teaching, some did not. The online education practice/wide range of teaching tools was usually utilized during the pursuit of a master’s degree. The individuals with a master’s degree showed a relatively less reduction in their commitment levels in comparison to those who only had an undergraduate degree. A reason for this finding can be due to the initial preparedness among the individuals owing to their educational background. Another reason for the global reduction in the commitment levels of teachers can be the discontinuation of online teaching platforms. Significant efforts were put by the teachers into these online teaching tools, and suddenly, all the efforts were abandoned. According to the study of Zhu and Liu (2020), the platforms for online teaching should evolve with the users, and they should be utilized for prolonged periods, even after the end of the pandemic. Unal and Bulunuz (2020) observed teachers demanding the online teaching platforms to remain parallel with face-to-face teaching in the post-lockdown period. However, a majority of schools worldwide have put an end to online teaching platforms since the start of face-to-face teaching. The same situation also applies to the schools where this study was applied to teachers.

The investigation done specifically on the school type showed disaggregation of teachers in terms of their commitment levels. Secondary school teachers showed the maximum drop in commitment levels as part of the transition. This can be linked to the difficulties that arise when younger students were asked to follow the COVID-19 measures (e.g., social distancing and wearing a mask). The stress levels of teachers may have an influence on their commitment levels, and this aspect should be extensively studied to develop a better understanding. *Post-hoc* analysis in this study also supports the idea that secondary school teachers show significant differences in commitment levels when compared to other teachers.

## Conclusion and Recommendations

This study was carried out to investigate the effects of two variables on the organizational commitment of teachers working in secondary educational institutions before and after the COVID-19 school lockdown in Cyprus. A quantitative methodology was employed in this study. In this quantitative study, an evaluation based on the demographics of the participants was carried out. In general, the pandemic resulted in an overall reduction in the commitment levels. Continuance commitment was the most influenced subcomponent, as its level reduced by 18%, followed by affective commitment, which saw a 10% drop. The reduction in normative commitment was negligible. When the demographic results were investigated, it was observed that gender differences had no significant effect on the results. However, a relative reduction in the commitment levels was observed. While it was initially determined that the commitment levels of married teachers were higher than those of single individuals, the post-pandemic results showed the opposite results. Postgraduate education had a positive impact on the post-lockdown commitment levels of teachers. Also, college teachers were observed to have the least reduction in commitment levels, while secondary school teachers were observed to have the highest reduction in commitment levels. Finally, having analyzed the time spent by teachers in the same school, it was found that, as the time spent in the same school increased, the commitment level also increased during the pre-pandemic lockdown period. However, this trend reversed during the post-pandemic lockdown period, where the commitment level of teachers decreased with the increase in the time spent in the same school. It is believed that this research will contribute to future studies on the organizational commitment of teachers, especially in the post-pandemic era.

As a result of the iterative model research, the recommendations presented below are expected to have a positive effect on the sustainable commitment levels of teachers.

1. The study was limited to a forced-choice response questionnaire and included a quantitative investigation. It is suggested that future studies should incorporate qualitative methods of data collection, so that the specific issues can be monitored thereon.

2. During the early times of the post-school lockdown period, teachers faced several situations that might have influenced their commitment levels. Studying these environmental factors in terms of demographic scale will allow decision-makers to improve the commitment levels of teachers at the level of specific groups.
3. Research should be carried out on the factors that keep the commitment levels of college teachers at good levels, and these methods should be modified such that they can be used for other school types to increase the commitment levels of teachers.
4. As the job experience of the teachers generally increased, a decrease in commitment level was observed. Also, as the time spent by the teachers in the same school increased, a positive effect was recorded. Planning job rotations or allowing elder teachers to carry out distance teaching may have a positive effect on the commitment levels.
5. The study revealed that the teachers who hold only a bachelor’s degree have a significant reduction in their commitment levels as a result of the implementation of COVID 19 precautions at schools. Further study options may have a positive impact on the commitment levels of teachers.

## Data Availability Statement

The original contributions presented in the study are included in the article/Supplementary Material, further inquiries can be directed to the corresponding author.

## Ethics Statement

This study was initially assessed and approved by European University of Lefke, Ethics Committee prior to the application of the survey on teachers. Written informed consent for participation was not required for this study in accordance with the national legislation and the institutional requirements.

## Author Contributions

IA performed the initial analyses and wrote the manuscript. OS assisted in the data collection and data analysis. Both authors contributed to the article and approved the submitted version.

## Conflict of Interest

The authors declare that the research was conducted in the absence of any commercial or financial relationships that could be construed as a potential conflict of interest.

## Publisher’s Note

All claims expressed in this article are solely those of the authors and do not necessarily represent those of their affiliated organizations, or those of the publisher, the editors and the reviewers. Any product that may be evaluated in this article, or claim that may be made by its manufacturer, is not guaranteed or endorsed by the publisher.
