# Supplementary material for: COVID-19 Impact on Teachers’ Organizational Commitment in Schools
Source: Front Psychol. 2022 May 31;13:810015. doi: 10.3389/fpsyg.2022.810015 (PMC9193219; doi:10.3389/fpsyg.2022.810015)
Supplement: Supplementary file 1 [file Data_Sheet_1.PDF]

Appendix 1. Organizational Commitment Scale (Rank between 1-5)

| | |
|---|---|
| Affective Commitment | I would be very happy to spend the rest of my career with this organization. |
| | I really feel as if this organization's problems are my own. |
| | I do not feel a strong sense of "belonging" to my organization. (R) |
| | I do not feel "emotionally attached" to this organization. (R) |
| | I do not feel like "part of the family" at my organization. (R) |
| | This organization has a great deal of personal meaning for me |
| Continuance Commitment | Right now, staying with my organization is a matter of necessity as much as desire. |
| | It would be very hard for me to leave my organization right now, even if I wanted to. |
| | Too much of my life would be disrupted if I decided I wanted to leave my organization now. |
| | I feel that I have too few options to consider leaving this organization. |
| | If I had not already put so much of myself into this organization, I might consider working elsewhere. |
| | One of the few negative consequences of leaving this organization would be the scarcity of available alternatives. |
| Normative Commitment | I do not feel any obligation to remain with my current employer. (R) |
| | Even if it were to my advantage, I do not feel it would be right to leave my organization now. |
| | I would feel guilty if I left my organization now. |
| | I would not leave my organization right now because I have a sense of obligation to the people in it. |
| | I would feel guilty if I left my organization now. |
| | I owe a great deal to my organization. |
